# Overexpression of Laminin 5*γ*2 Chain Correlates with Tumor Cell Proliferation, Invasion, and Poor Prognosis in Laryngeal Squamous Cell Carcinoma

**DOI:** 10.1155/2022/7248064

**Published:** 2022-10-15

**Authors:** Chengyi Yin, Bingliang Ma, Xilin Zhang, Longjiang Lan, Gang Ren, Jue Xu, Jianqiu Wang

**Affiliations:** ^1^Department of Otolaryngology, The First Affiliated Hospital, Huzhou University, The First People's Hospital of Huzhou, Huzhou City, Zhejiang Province, China; ^2^Research Department, The First Affiliated Hospital, Huzhou University, The First People's Hospital of Huzhou, Huzhou City, Zhejiang Province, China

## Abstract

**Objective:**

Laryngeal squamous cell carcinoma (LSCC) is a common malignant tumor. Laminin 5*γ*2 chain (LAMC2) was reported to be associated with tumorigenesis. This study explored the role of LAMC2 on LSCC progression by regulating the integrin*β*1/FAK/Src/AKT pathway.

**Methods:**

The level of LAMC2 in 46 LSCC patients was detected by qRT-PCR and western blot. Then the relationship between LAMC2 expression and LSCC malignancy as well as prognosis was analyzed, and the effect of LAMC2 expression on LSCC patient survival was also analyzed using the Kaplan–Meier survival curves. Afterwards, the LSCC cells were transfected with LAMC2 overexpression and knockdown vectors, the effect of LAMC2 on LSCC cell viability, proliferation ability, cell cycle, cell migration, and invasion were detected by CCK-8, colony formation, flow cytometry, wound healing, and Transwell assays. The expression of EMT-related biomarkers and integrin *β*1/FAK/Src/AKT signaling-related proteins was detected by western blot. Moreover, the effect of LAMC2 on LSCC tumor growth was evaluated by *in vivo* xenograft experiments and western blot.

**Results:**

LAMC2 was expressed at high level in LSCC tissues and associated with poor prognosis. LAMC2 overexpression increased TU177 cell viability, proliferation ability, promoted cell cycle, cell migration, and invasion capacity. The expression of N-cadherin, vimentin, and integrin*β*1/FAK/Src/AKT related proteins was increased, while the expression of E-cadherin protein was decreased. When the LAMC2 knockdown in AMC-HN-8 cells had opposite effects. Furthermore, shLAMC2 decreased tumor volume and the expression of LAMC2, Ki-67 and integrin*β*1, but increased the expression of E-cadherin in LSCC tumor-bearing mice.

**Conclusion:**

The findings suggested that LAMC2 was overexpressed in LSCC and correlated with poor prognosis. LAMC2 knockdown inhibited LSCC progression by regulating the integrin*β*1/FAK/Src/AKT signaling pathway. Therefore, LAMC2 could be a target for LSCC therapy.

## 1. Introduction

Laryngeal cancer is a malignant tumor occurred at the larynx, accounting for 5% of the malignant tumor of human body, of which more than 95% is laryngeal squamous cell carcinoma (LSCC) [1]. LSCC is one of the most common tumors in the head and neck [2]. The main causes of LSCC are smoking and drinking, and more than 95% of LSCC patients have a history of long-term and heavy smoking and (or) drinking [3]. Clinical manifestations include hoarseness, dyspnea, dysphagia, and cervical lymph node metastasis [4]. LSCC has a high morbidity and mortality [5]. Epidemiological surveys found that the global incidence of laryngeal cancer increased by 12% in the past 30 years [6]. Surgical treatment of LSCC often requires total laryngectomy, which seriously threatens the quality of life of patients [7]. Cancer recurrence and metastasis are the most important factors for the prognosis of patients with LSCC [8]. In the past 20 years, despite advances in chemotherapy and radiotherapy, the survival rate of patients has not improved [9], and the overall survival rate for laryngeal cancer patients was found to be increasing [10]. Therefore, understanding the molecular mechanisms of LSCC carcinogenesis is urgently needed to develop more effective therapeutic strategies.

LAMC2 (Laminin 5*γ*2 chain, LN-5*γ*2) is a high molecular weight protein of the extracellular matrix that is a heterotrimeric protein [11]. LAMC2 has been implicated in the pathogenesis of cancers in increasing numbers of studies [12, 13]. In addition, LAMC2 is overexpressed in various cancers [14–16]. Gao et al. compared expression profiles of genes in paired adjacent normal mucosal tissue and LSCC tissues by RNA-sequencing transcriptome sequencing and found that LAMC2 was significantly up-regulated in LSCC tissues [17]. However, the role of LAMC2 in the pathogenesis of LSCC remains to be elucidated.

Integrins are heterodimers composed of *α* and *β* subunits on the cell surface, which have the functions of adhesion and signal transduction [18]. They are expressed in tumors and promotes tumor proliferation and migration [19]. Focal adhesion kinase (FAK) is a nonreceptor intracellular tyrosine kinase that is an important member of integrin-mediated signaling [20]. Integrin*β*1 receptors regulate multiple signaling pathways, such as FAK/AKT or FAK/ERK signaling pathways [21]. Src is a nonreceptor tyrosine kinase whose expression is elevated in various cancers [22]. The *β*1 integrin-mediated pathway relies on Src kinase and PI3K [23]. In addition, it has been found that LAMC2 induced cancer cell invasion by interacting with specific receptors and activating signaling mediators such as FAK and AKT [24]. Liang et al. found that increasing LAMC2 expression could stimulate the FAK-PI3K/AKT signaling pathway to accelerate esophageal squamous cell carcinoma (ESCC) metastasis [15]. In addition, epithelial-mesenchymal transition (EMT) is thought to contribute to tumor progression, and aberrant expression of EMT regulator/inducers in cancer cells correlates with tumor aggressiveness and poor clinical outcomes [25]. Recent studies have reported that LAMC2 is frequently overexpressed in cancer cells, particularly that have undergone EMT in different cancer types [26–28]. However, it is unclear whether the occurrence of LSCC is related to the regulation of integrin*β*1/FAK/Src/AKT by LAMC2.

In the study, it was found that LAMC2 was overexpressed in LSCC and correlated with poor prognosis. LAMC2 promoted the proliferation and invasiveness in LSCC by regulating the integrin*β*1/FAK/Src/AKT signaling pathway. The findings suggested that LAMC2 could be an important indicator of LSCC progression and poor survival. In addition, the innovation of this study lied in the collection of clinical information and samples for testing, as well as mouse tumor-bearing experiments to explore the correlation between LAMC2 overexpression and LSCC proliferation, invasion, and prognosis.

## 2. Materials and Methods

### 2.1. Patients and Tissue Samples

Fresh LSCC and adjacent nontumor tissues (5 cm from the tumor boundary) were collected from 46 LSCC patients who underwent surgical resection in the Department of Otolaryngology, the First People's Hospital of Huzhou (Zhejiang, China), from January 2018 to December 2020. In patients and tissue samples, the method to obtain the clinical baseline in Table 1 was a self-made questionnaire. The patient did not receive any treatment before admission. All tissue specimens were histopathologically examined in our hospital, and the eighth edition of the American Joint Committee on Cancer (AJCC) tumor-lymph node metastasis (TNM) staging system was used [29]. The samples were stored at −80°C. The study was approved by the Ethics Committee of the First People's Hospital of Huzhou (Zhejiang, China), and informed consent was obtained from each participant.

### 2.2. Cell Culture and Plasmid Transfection

LSCC cells lines (TU177, AMC-HN-8, TU686, and TU212) were purchased from Cyberkang (Shanghai) Biotechnology Co., Ltd. Human bronchial epithelial cells (16HBE) cells were obtained from ATCC (Manassas, VA, USA). LSCC cells and 16HBE cells were cultured in DMEM high glucose medium (Hyclone, SH30243.01) containing fetal bovine serum (FBS) (Zhejiang Tianhang Biological Technology Co., Ltd., 11011–8615) at 37°C and 5% CO_2_. When the cell growth density reached 80%, the old medium was discarded, digested with 0.25% trypsin (Hyclone, SH30042.01), and resuspended by adding a fresh medium. TU177 cells were transfected with empty vector and LAMC2 plasmids for overexpression experiments. Then AMC-HN-8 cells were transfected with shNC, shLAMC2#1, and shLAMC2#2 for knockdown experiments. In this research, plasmids were transfected into cells using transfection reagent Lipofectamine 2000.

### 2.3. Quantitative Real Time PCR (qRT-PCR)

Cells or tissues were lysed with Trizol reagent (Vitality, B511311) and total RNA was extracted. Synthesis of cDNA by reverse transcription was performed, and then the cDNA was analyzed by SYBR Premix Ex TaqII (Takara, RR820A). The level of the target gene was calculated by 2^−△△Ct^ method [30], and GAPDH was used as the internal reference. The gene primer sequences are shown in Table 2.

### 2.4. CCK8 Assay

Cells in logarithmic growth phase were cultured in 96-well plates for 24 h. Then CCK-8 solution (Biyuntian Biotechnology Co., Ltd., Shanghai, China, C0039) was added and incubated for 2 h. Finally, the absorbance at 450 nm was measured, and the cell viability was calculated.

### 2.5. Colony Formation Assay

TU177 cells were divided into the vector and LAMC2 group, and the AMC-HN-8 cells were divided into the shNC and shLAMC2 group. Then the cells in log phase were trypsinized, and 500 cells/well were seeded in a complete medium containing 30% FBS (Hyclone, SH30256.01) and incubated. The medium was changed every 3 days and the cell status was observed. Then 1000 *μ*L of impurity-free crystal violet staining solution (Shanghai Qiangshun Chemical Reagents Co., Ltd., 548-62-9) was added to stain cells. The size of the clone was observed under a microscope (Motic, AE2000), and a photograph can be taken. Colonies consisting of >50 cells were counted.

### 2.6. Flow Cytometry

The cell suspensions of the TU177 and AMC-HN-8 cells in the logarithmic phase were inoculated into the 6-well plates and were precultured for 24 h, as described in the section “Cell cultures and treatment.” After 24 h of administration, the cells in each cell cycle were detected by flow cytometry.

### 2.7. Wound Healing Assay

Cell migration was detected by a wound-healing assay. Approximately 5 × 10^5^ cells were added to each well and incubated overnight. The next day, cells were washed 3 times to remove dead cells and incubated with a serum-free medium. The wound healing distance was measured by taking pictures at 0 h and 24 h, respectively.

### 2.8. Transwell Assay

TU177 cells were divided into the vector and LAMC2 group, and the AMC-HN-8 cells were divided into the shNC and shLAMC2 group. After 24 h of treatment, Transwell chambers (Corning, 3422) were placed in 24-well plates for culture. The serum-free DMEM medium was diluted and incubated overnight in Transwell chambers. Crystal violet staining was used, photographs were taken, and the number of migrated and invasive cells was counted.

### 2.9. Western Blot

Tissue proteins were prepared and the total protein concentration was measured. Then SDS-PAGE electrophoresis was performed and the membrane was transferred by transfer membrane (TianGen, Beijing, China, VE186). Then the primary antibodies including anti-LAMC2 (DF9052), anti-E-cadherin (AF0131), anti-N-cadherin (AF4039), pan-AKT1/2/3 (AF6259), phospho-pan-AKT1/2/3 (AF0016), phospho-FAK (AF3398), anti-FAK (AF6397), anti-c-Myc (AF0358), anti-cyclin D1 (AF0931), anti-cyclin E (AF0144), anti-CDK2 (AF6237), anti-CDK4 (DF6102), anti-MMP9 (AF5228), phospho-Src (AF3162), anti-Src (AF6162), anti-ZEB1 (DF7414), anti-vimentin (AF7013), anti-integrin *β*1 (AF5379), Ki-67 (AF0198), and GAPDH (AF7021) (all antibodies were purchased from Affinity) were put in at a ratio of 1 : 1500, and then they were cut open to incubate the antibody. The membrane was rinsed and the secondary antibody (HRP) (ab97080, 1 : 2000, Abcam) was added. The ECL was used to detect protein bands, and the protein gray value was calculated by Image J (NIH Image J system, Bethesda, MD).

### 2.10. Xenograft Tumor Experiments

For animal xenograft model assays, 3 × 10^6^ AMC-HN-8 cells were injected subcutaneously into the posterior flanks of the 6-week-old BALB/c nude mice (weight about 30 g) from Beijing Vital River Laboratory Animal Technology Co., Ltd, and the license number is SCXK (Beijing) 2016–0011. Then tumor-bearing mice were randomly assigned to shNC and shLAMC2 groups (*n* = 6). Tumor diameters were measured 7 days after injection. At 31 days, mice were sacrificed and dissected, and tumor tissue was weighed. The tumor volume was calculated as follows: tumor volume = (length × width^2^)/2 [31].

### 2.11. Statistical Analysis

SPSS 22.0 was used to analyze the data. If the measurement data between multiple groups conformed to the normal distribution and conformed to the homogeneity of variance test, one-way-ANOAY analysis of variance was used, and the Tukey test was used for further pairwise comparisons between the groups. The measurement data were compared between the two groups using the student's *t* test. Homogeneity of variances is tested by using Levene's, if the variances are homogeneous, refer to the statistics under “Assume equal variances”; if the variances are unequal, refer to the statistics under “Do not assume equal variances.” The enumeration data were compared using the *χ*^2^ test, and the survival rate was using the Kaplan–Meier method. The data were expressed as mean ± standard deviation (SD), *P* < 0.05 was statistically significant.

## 3. Results

### 3.1. LAMC2 was Highly Expressed in LSCC and Associated with Poor Prognosis

**Figure 1:**
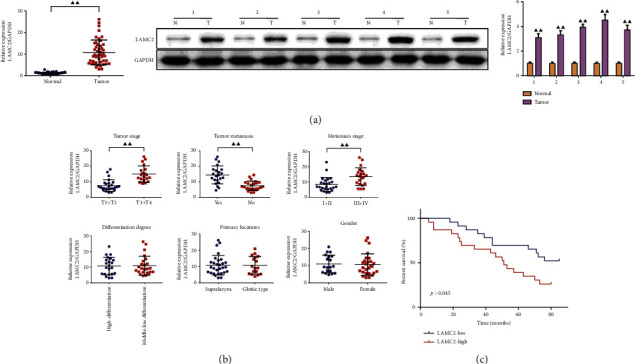
LAMC2 was highly expressed in LSCC and associated with poor prognosis. Expression of LAMC2 in LSCC patients by qRT-PCR and Western blot (*n* = 3) (a), relationship between LAMC2 expression and clinicopathologic parameters of LSCC patients (*n* = 23) (b) and Kaplan–Meier method and log-rank analysis of the overall survival rate of patients with high LAMC2 expression (*n* = 23) (c). Data were expressed as mean ± SD. Compared to the normal group, ^▲^*P* < 0.05, ^▲▲^*P* < 0.01.

The mRNA expression of LAMC2 in 46 LSCC patients was detected by qRT-PCR, and the protein expression of LAMC2 in five pairs of samples was detected by western blot. The results showed that the expression of LAMC2 was significantly increased in tumor tissues compared with the normal tissues (Figure 1(a) and Supplementary files Figure 1).

To determine the relationship between LAMC2 expression and LSCC malignancy, patients were divided into LAMC2-low (below the median, *n* = 23) and LAMC2-high (above the median, *n* = 23) expression groups by the relative expression of LAMC2 mRNA. As shown in Table 1 and Figure 1(b), the high expression of LAMC2 had statistical significance with advanced tumor stages, and advanced tumor lymph node metastasis stages. However, LAMC2 expression was independent of clinical variables such as gender, tumor differentiation, and primary site.

The overall in survival difference between the low-expression group and high-expression group of LAMC2 was compared by the survival curves, and the clinical significance of the expression of LAMC2 in LSCC was further evaluated, as shown in Figure 1(c). Kaplan–Meier method and log-rank test found that the LAMC2-high expression group died in the fifth month, while patients died in the 18th month in the LAMC2-low expression group. The overall survival rate of patients with high LAMC2 expression was significantly lower than that of patients with low LAMC2 expression.

### 3.2. LAMC2 was Highly Expressed in LSCC Cells

**Figure 2:**
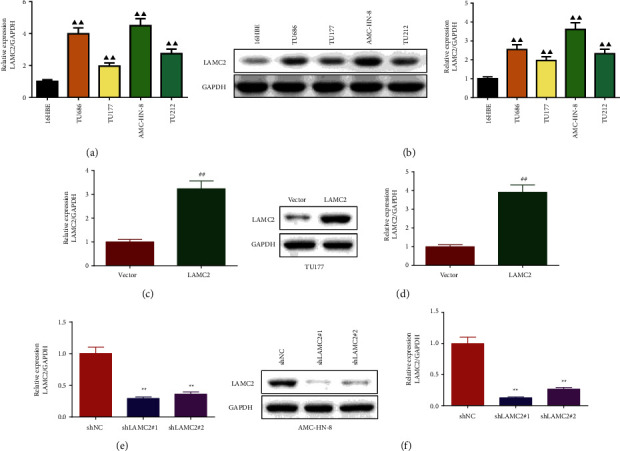
LAMC2 was highly expressed in LSCC cells. (a) and (b): LAMC2 mRNA and protein expression in LSCC cells texted by qRT-PCR and western blot. (c) and (d): qRT-PCR and western blot were performed to detect the effect of LAMC2 overexpression on LAMC2 mRNA and protein expression. (e) and (f): LAMC2 knockdown inhibited the expression of LAMC2 mRNA and protein in AMC-HN-8 cells. qRT-PCR and Western blot were performed to detected the effect of LAMC2 knockdown on LAMC2 mRNA and protein expression. Data were expressed as mean ± SD, *n* = 3. Compared to the 16HBE group, ^▲▲^*P* < 0.01. Compared to the vector group, ^##^*P* < 0.01. Compared to the shNC group, ^∗∗^*P* < 0.01.

**Figure 3:**
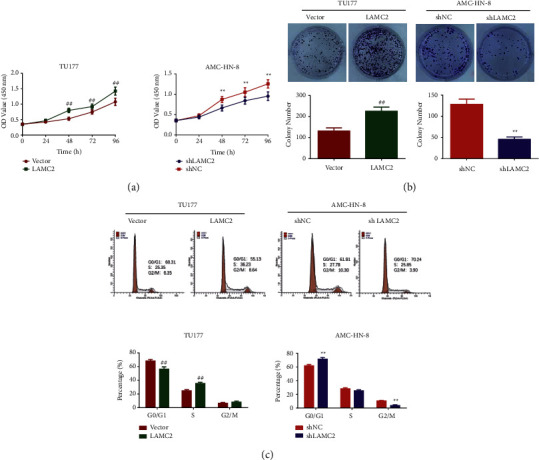
LAMC2 promoted the proliferation and cell cycle of LSCC cells. (a) LAMC2 increased AMC-HN-8 and TU177 cell viability. CCK8 assay was performed to detect the effect of LAMC2 overexpression and knockdown on LSCC cell viability. (b) LAMC2 facilitated the colony formation of AMC-HN-8 and TU177 cells. Colony formation assay was performed to detect the effect of LAMC2 overexpression and knockdown on LSCC cell proliferation. (c) LAMC2 reduced G0/G1 phase in AMC-HN-8 and TU177 cells. Flow cytometry was performed to detect the effect of LAMC2 overexpression and knockdown on LSCC cell cycle. Data were expressed as mean ± SD, *n* = 3. Compared to the vector group, ^##^*P* < 0.01. Compared to the shNC group, ^∗∗^*P* < 0.01.

**Figure 4:**
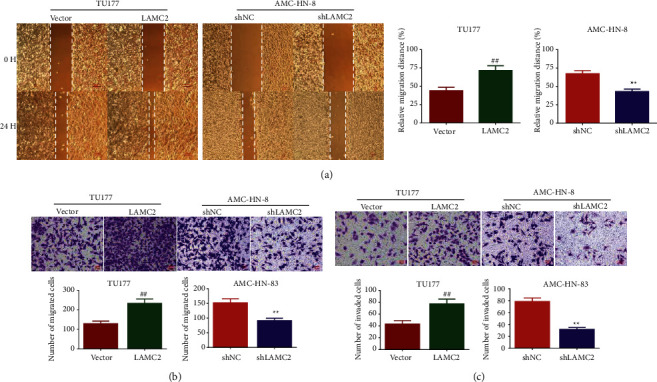
LAMC2 promoted TU177 and AMC-HN-8 cells migration and invasion. (a)–(b): LAMC2 promoted TU177 and AMC-HN-8 cells migration. Wound healing and Transwell assays were performed to detect the effect of LAMC2 overexpression and knockdown on LSCC cell migration. (c) LAMC2 promoted TU177 and AMC-HN-8 cells invasion. Transwell assay was performed to detect the effect of LAMC2 overexpression and knockdown on LSCC cell invasion. Data were expressed as mean ± SD, *n* = 3. Compared to the vector group, ^##^*P* < 0.01. Compared to the shNC group, ^∗∗^*P* < 0.01.

As can be seen from Figures 2(a) and 2(b) and Supplementary files Figure 2, compared with the 16HBE, the levels of LAMC2 mRNA and protein in LSCC cells, including TU686, TU177, AMC-HN-8, and TU212 were significantly increased. As TU177 cells had the lowest expression of LAMC2, overexpression experiments of LAMC2 were performed on TU177 cells, and the results are shown in Figures 2(c) and 2(d) and Supplementary files Figure 3. Compared with the vector group, LAMC2 mRNA and protein levels were higher in the LAMC2 group. As AMC-HN-8 cells had the most expression of LAMC2, knockdown experiments of LAMC2 were performed on AMC-HN-8 cells and the results were shown in Figures 2(e) and 2(f) and Supplementary files Figure 4. LAMC2 mRNA and protein levels in the shLAMC2#1 and shLAMC2#2 groups were lower than those in the shNC group. Since the LAMC2 mRNA and protein in the shLAMC2#1 group were lower, the shLAMC2#1 group was selected for subsequent experiments.

### 3.3. LAMC2 Promoted the Proliferation and Cell Cycle of LSCC Cells

As shown in Figure 3(a), compared to the relevant control group, the cell viability in the LAMC2 group was higher than the shLAMC2 group at 48–96 h. The results of cell colony formation ability were shown in Figure 3(b). The cell colony formation ability in the LAMC2 group was increased. However, the colony formation ability of cells in the shLAMC2 group was decreased. The cell cycle results are shown in Figure 3(c), the percentages of cells were decreased in G0/G1 phase, and the percentages of cells in S phase were increased in the LAMC2 group. In the shLAMC2 group, the percentages of cells in G0/G1 phase were increased, while the percentages of cells in G2/M phase were decreased.

### 3.4. LAMC2 Promoted LSCC Cells Migration and Invasion

The wound healing assay results are shown in Figure 4(a). The migration rate of cells in the LAMC2 group was increased, and the migration ability was enhanced. However, the migration rate of cells in the shLAMC2 group decreased, and the migration ability weakened. The effect of LAMC2 on the migration ability of cells was further tested by cell migration assay and the result is shown in Figure 4(b). The number of migrated cells in the LAMC2 group was higher than that in the vector group. The number of migrated cells in the shLAMC2 group was lower than that in the shNC group. The effect of LAMC2 on the number of invaded cells is shown in Figure 4(c). Relative to the vector group, the number of invaded cells of TU177 in the LAMC2 group was enhanced. Relative to the shNC group, the number of invaded cells of AMC-HN-8 in the shLAMC2 group was decreased.

### 3.5. LAMC2 Promoted the EMT in LSCC Cells

**Figure 5:**
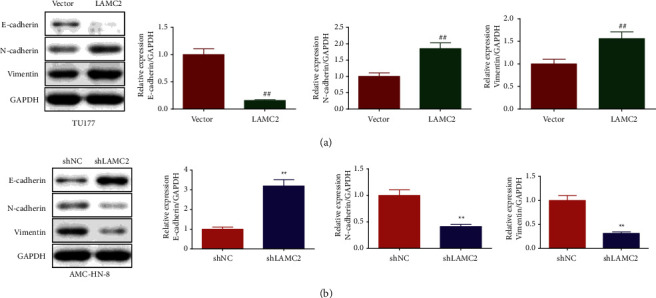
LAMC2 promoted the EMT in LSCC cells. (a) Overexpression of LAMC2 promoted the expression of N-cadherin and vimentin proteins in TU177 cells and inhibited the expression of E-cadherin protein. Western blot was performed to detect the effect of LAMC2 overexpression on the EMT in LSCC cells. (b) LAMC2 knockdown inhibited N-cadherin and vimentin protein expression and promoted E-cadherin protein expression in AMC-HN-8 cells. Western blot was performed to detect the effect of LAMC2 knockdown on the EMT in LSCC cells. Data were expressed as mean ± SD, *n* = 3. Compared to the vector group, ^##^*P* < 0.01. Compared to the shNC group, ^∗∗^*P* < 0.01.

**Figure 6:**
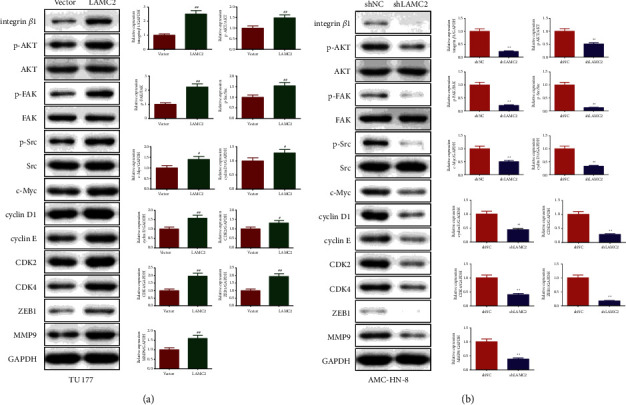
LAMC2 activated the integrin *β*1/FAK/Src/AKT signaling pathway in TU177 and AMC-HN-8 cells. (a) LAMC2 overexpression promotes integrin *β*1/FAK/Src/AKT protein expression in TU177 cells. (b) LAMC2 knockdown inhibited integrin *β*1/FAK/Src/AKT protein expression in AMC-HN-8 cells. Data were expressed as mean ± SD, *n* = 3. Compared to the vector group, ^#^*P* < 0.05, ^##^*P* < 0.01. Compared to the shNC group, ^∗^*P* < 0.05, ^∗∗^*P* < 0.01.

The effect of LAMC2 on the EMT progress in LSCC cells migration and invasion was also assessed by detecting the expression of N-cadherin, vimentin, and E-cadherin. As seen in Figure 5(a) and Supplementary files Figure 5, relative to the vector group, the level of E-cadherin was decreased in the LAMC2 group, while the levels of N-cadherin and vimentin were increased in the LAMC2 group. As shown in Figure 5(b) and Supplementary files Figure 6, compared with the shNC group, the level of E-cadherin in the shLAMC2 group was increased, while the levels of N-cadherin and vimentin were decreased in the shLAMC2 group.

### 3.6. LAMC2 Activated the integrin*β*1/FAK/Src/AK Signaling Pathway in LSCC Cells

**Figure 7:**
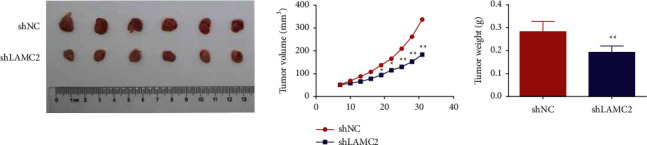
Knockdown of LAMC2 inhibited tumor growth in AMC-HN-8 cell xenograft mice. Data were expressed as mean ± SD, *n* = 6. Compared to the shNC group, ^∗^*P* < 0.05, ^∗∗^*P* < 0.01.

**Figure 8:**
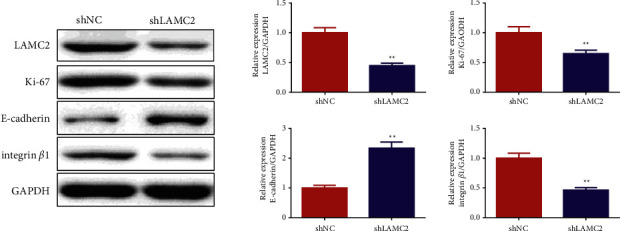
Knockdown of LAMC2 reduced the expression of LAMC2, Ki-67, and integrin*β*1, while increased the expression of E-cadherin. Data were expressed as mean ± SD, *n* = 3. Compared with the shNC group, ^∗^*P* < 0.05, ^∗∗^*P* < 0.01.

The effect of LAMC2 on integrin*β*1/FAK/Src/AKT signaling pathway-related proteins in TU177 cells is shown in Figure 6(a) and Supplementary files Figure 7. Compared with the vector group, the levels of integrin*β*1, p-AKT, p-FAK, p-Src, c-Myc, cyclin D1, cyclin E, CDK2, CDK4, ZEB1, and MMP9 proteins in the LAMC2 group were increased. Accordingly, in AMC-HN-8 cells (Figure 6(b) and Supplementary files Figure 8), compared to the shNC group, the expressions of integrin*β*1, p-AKT, p-FAK, p-Src, c-Myc, cyclin D1, cyclin E, CDK2, CDK4, ZEB1, and MMP9 proteins in the shLAMC2 group were reduced.

### 3.7. Knockdown of LAMC2 Inhibited Tumor Growth in AMC-HN-8 Cell Xenograft Mice

As can be seen from Figure 7, 19 days after inoculation, relative to the shNC group, the tumor volume in the shLAMC2 group was reduced. After 31 days of inoculation, the tumor mass in the shLAMC2 group was lower than that in the shNC group.

### 3.8. Knockdown of LAMC2 Reduced the Expression of LAMC2, Ki-67, and integrin*β*1

The effect of knockdown of LAMC2 on the expression of LAMC2, Ki-67, E-cadherin, and integrin*β*1 was investigated by western blot in tumor tissues of nude mice. The results are shown in Figure 8 and Supplementary files Figure 9. Compared with the shNC group, the expression of LAMC2, Ki-67, and integrin*β*1 was decreased in the shLAMC2 group, while the expression of E-cadherin was increased.

## 4. Discussion

LSCC is one of the most common malignant tumors [32]. The incidence is increasing, and the treatment efficacy and prognosis are not ideal [33]. Therefore, it is very important to understand the related mechanism of LSCC and improve the intervention measures for the treatment of LSCC. This study found that LAMC2 was highly expressed in LSCC and associated with poor prognosis. Furthermore, LAMC2 was found to regulate LSCC progression through the integrin*β*1/FAK/Src/AKT signaling pathway. Finally, knockdown LAMC2 inhibited LSCC cell xenograft tumor growth *in vivo*.

In this study, the expression of LAMC2 was up-regulated in fresh LSCC tissues of LSCC patients. However, LAMC2 expression was independent of clinical variables such as gender, tumor differentiation, and primary site. This finding was similar to the finding of Kirtonia et al. which showed that LAMC2 up-regulated in pancreatic ductal adenocarcinoma (PDAC) tumor specimens [34]. In addition, the levels of LAMC2 mRNA and protein in LSCC cells were higher than that in 16HBE. The LAMC2 mRNA and protein were decreased in the LAMC2 knockdown group, while increased in the overexpression group. Jin et al. found that transfection of LAMC2 overexpression vector in hepatocellular carcinoma HepG2 and Huh-7 cells promoted the expression of LAMC2 mRNA [35]. Moon et al. found that LAMC2 protein was increased in lung adenocarcinoma (ADC) cells in the LAMC2 overexpression group [26].

LAMC2 was found to promote the growth of LSCC cells by CCK-8, colony formation, and flow cytometric assays, which was similar to the findings of Wang et al., who found that LAMC2 promoted the proliferation and cell viability of laryngeal cancer cells [36]. Moreover, LAMC2 promoted LSCC cell migration and invasion. Ning et al. found that knockdown of LAMC2 inhibited the proliferation, colony formation, and migration of oral squamous cell carcinoma cells [37]. Furthermore, Zhou et al. showed that silencing LAMC2 inhibited the proliferation, migration, and invasion of OSCC cells [38]. Finally, LAMC2 promoted the expression of N-cadherin and vimentin proteins *in vitro*. Silencing LAMC2 in PDAC cells inhibited the expression of N-cadherin and vimentin, while induced the expression of E-cadherin [34].

EMT was generally associated with the invasion and migration ability of cells [39], increased cellular capacity to migrate and invade coincides with EMT [40]. EMT has been reported to be involved in the development of LSCC [41]. Li et al. observed that miR-625 overexpression inhibited EMT in LSCC cells by increasing the expression levels of E-cadherin and decreasing the expression levels of N-cadherin and vimentin [42]. Hong et al. found that EMT, proliferation, and invasion of renal cell carcinoma cells were inhibited by the Src/FAK signaling pathway [43].

In this study, overexpression of LAMC2 promoted the expression of proteins related to integrin*β*1/FAK/Src/AKT molecules, and knockdown of LAMC2 inhibited the expression of proteins related to integrin*β*1/FAK/Src/AKT molecules, which further proved that LAMC2 promoted cell proliferation and invasion through the integrin*β*1/FAK/Src/AKT signaling pathway. This was similar to what Abula et al. found in PDAC cells, where PDAC proliferation and invasion were promoted by activating the Src/FAK/AKT signaling pathway [44]. Furthermore, Guo et al. found that Src/Fak, Akt, and Erk1/2 signaling regulate proliferation and migration of gastric cancer cells [45]. Knockdown of LAMC2 inhibited LSCC cell tumor growth *in vivo* in xenograft experiments. Zhou et al. found that silencing LAMC2 inhibited the growth of subcutaneously transplanted tumors in nude mice [38].

However, this study also has certain limitations. Due to the insufficient sample size, the effect of LAMC2 regulating integrin *β*1/FAK/Src/AKT signaling pathway in improving LSCC in this study was not sufficient. Future studies with larger samples are needed to verify the mechanism of action of LAMC2 on LSCC improvement.

## 5. Conclusion

In conclusion, this study found that LAMC2 was overexpressed in LSCC tissues and correlated with poor prognosis of patients. Overexpression of LAMC2 promoted the migration and invasion of LSCC cells. This study demonstrated the role of LAMC2 in activating the integrin*β*1/FAK/Src/AKT signaling pathway in LSCC, promoting LSCC cell proliferation and invasion. Thus, the significance of this study is that modulating the expression of LAMC2 could be a target for LSCC therapy.

## Figures and Tables

**Table 1:** Relationship between LAMC2 expression and clinicopathologic parameters of LSCC patients.

Characteristic	Number of cases	LAMC2-low expression group (*n* = 23)	LAMC2-high expression group (*n* = 23)	*χ* ^2^	*P*-value
Gender					
Male	19	10	9	0.09	0.765
Female	27	13	14		

Primary site					
Supralarynx	29	14	15	0.093	0.760
Glottic type	17	9	8		

Tumor stage					
T1 + T2	26	21	5	22.646	0.000
T3 + T4	20	2	18		

The degree of differentiation					
High differentiation	22	9	13	1.394	0.238
Low/moderate differentiation	24	14	10		

Lymph node metastasis					
Yes	22	5	17	12.545	0.000
No	24	18	6		

TNM stage					
I + II	25	17	8	7.097	0.008
III + IV	21	6	15		

**Table 2:** qRT-PCR Primer sequences.

Gene	Forward primer (5′–3′)	Reverse primer (5′–3′)
Human LAMC2	AGGAGCAGAAGCTTTCCC	TGAATGGGCCTGCCTTACAG
Human GAPDH	AATGGGCAGCCGTTAGGAA	GCGCCCAATACGACCAAATC

## Data Availability

All data generated or analyzed during this study are included in this published article.

## Supplementary Materials

Supplementary Materials The supplementary materials are the as full as possible length gels blots and the original images. Supplementary files Figure 1. The LAMC2 and GAPDH protein bands are corresponding the original band of Figure 1A in the manuscript. Supplementary files Figure 2. The LAMC2 and GAPDH protein bands are corresponding the original band of Figure 2B in the manuscript. Supplementary files Figure 3. The LAMC2 and GAPDH protein bands are corresponding the original band of Figure 2D in the manuscript. Supplementary files Figure 4. The LAMC2 and GAPDH protein bands are corresponding the original band of Figure 2F in the manuscript. Supplementary files Figure 5. The E-cadherin, N-cadherin, vimentin, and GAPDH protein bands are corresponding the original band of Figure 5A in the manuscript. Supplementary files Figure 6. The E-cadherin, N-cadherin, vimentin, and GAPDH protein bands are corresponding the original band of Figure 5B in the manuscript. Supplementary files Figure 7. The integrin*β*1, p-AKT, AKT, p-FAK, FAK, p-Src, src, c-Myc, cyclin D1, cyclin E, CDK2, CDK4, ZEB1, MMP9, and GAPDH protein bands are corresponding the original band of Figure 6A in the manuscript. Supplementary files Figure 8. The integrin*β*1, p-AKT, AKT, p-FAK, FAK, p-Src, src, c-Myc, cyclin D1, cyclin E, CDK2, CDK4, ZEB1, MMP9, and GAPDH protein bands are corresponding the original band of Figure 6B in the manuscript. Supplementary files Figure 9. The LAMC2, Ki-67, E-cadherin, and integrin*β*1 protein bands are corresponding the original band of Figure 8 in the manuscript.
