# Supplementary material for: Overexpression of Laminin 5γ2 Chain Correlates with Tumor Cell Proliferation, Invasion, and Poor Prognosis in Laryngeal Squamous Cell Carcinoma
Source: J Oncol. 2022 Oct 15;2022:7248064. doi: 10.1155/2022/7248064 (PMC9588344; doi:10.1155/2022/7248064)
Supplement: Supplementary Materials — The supplementary materials are the as full as possible length gels blots and the original images. Supplementary files Figure 1. The LAMC2 and GAPDH protein bands are corresponding the original band of Figure 1A in the manuscript. Supplementary files Figure 2. The LAMC2 and GAPDH protein bands are corresponding the original band of Figure 2B in the manuscript. Supplementary files Figure 3. The LAMC2 and GAPDH protein bands are corresponding the original band of Figure 2D in the manuscript. Supplementary files Figure 4. The LAMC2 and GAPDH protein bands are corresponding the original band of Figure 2F in the manuscript. Supplementary files Figure 5. The E-cadherin, N-cadherin, vimentin, and GAPDH protein bands are corresponding the original band of Figure 5A in the manuscript. Supplementary files Figure 6. The E-cadherin, N-cadherin, vimentin, and GAPDH protein bands are corresponding the original band of Figure 5B in the manuscript. Supplementary files Figure 7. The integrinβ1, p-AKT, AKT, p-FAK, FAK, p-Src, src, c-Myc, cyclin D1, cyclin E, CDK2, CDK4, ZEB1, MMP9, and GAPDH protein bands are corresponding the original band of Figure 6A in the manuscript. Supplementary files Figure 8. The integrinβ1, p-AKT, AKT, p-FAK, FAK, p-Src, src, c-Myc, cyclin D1, cyclin E, CDK2, CDK4, ZEB1, MMP9, and GAPDH protein bands are corresponding the original band of Figure 6B in the manuscript. Supplementary files Figure 9. The LAMC2, Ki-67, E-cadherin, and integrinβ1 protein bands are corresponding the original band of Figure 8 in the manuscript. [file 7248064.f1.pdf]

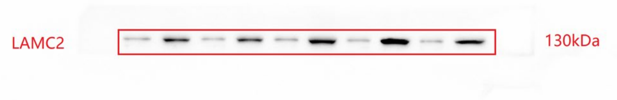

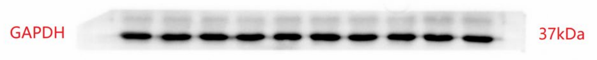

Supplementary files Fig 1. The LAMC2 and GAPDH protein bands are corresponding the original band of Fig. 1A in the manuscript.

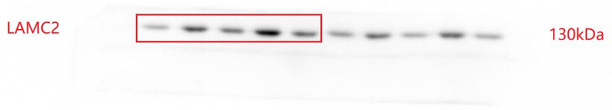

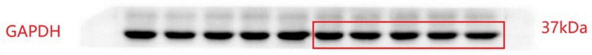

Supplementary files Fig 2. The LAMC2 and GAPDH protein bands are corresponding the original band of Fig. 2B in the manuscript.

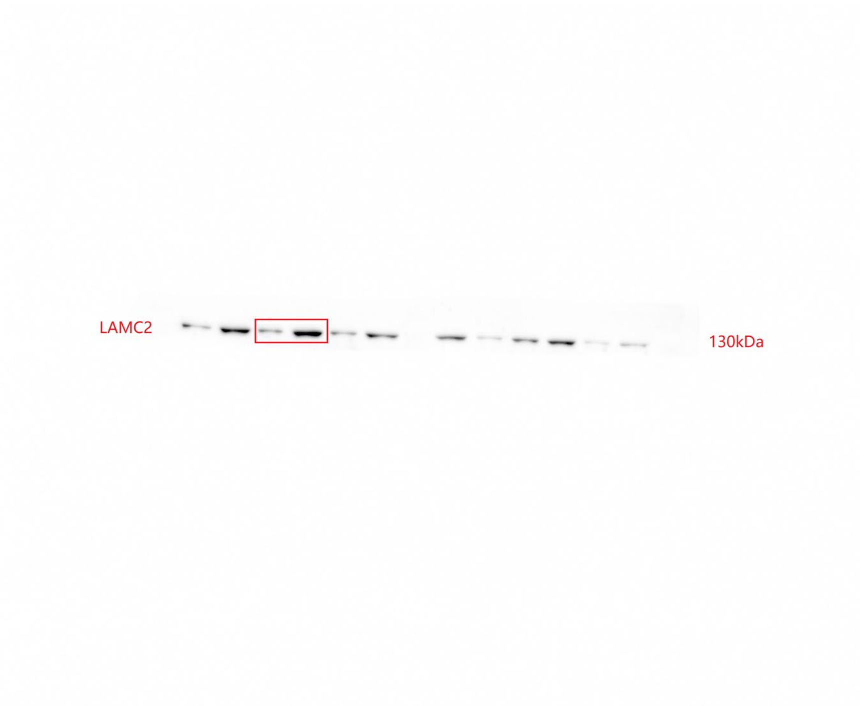

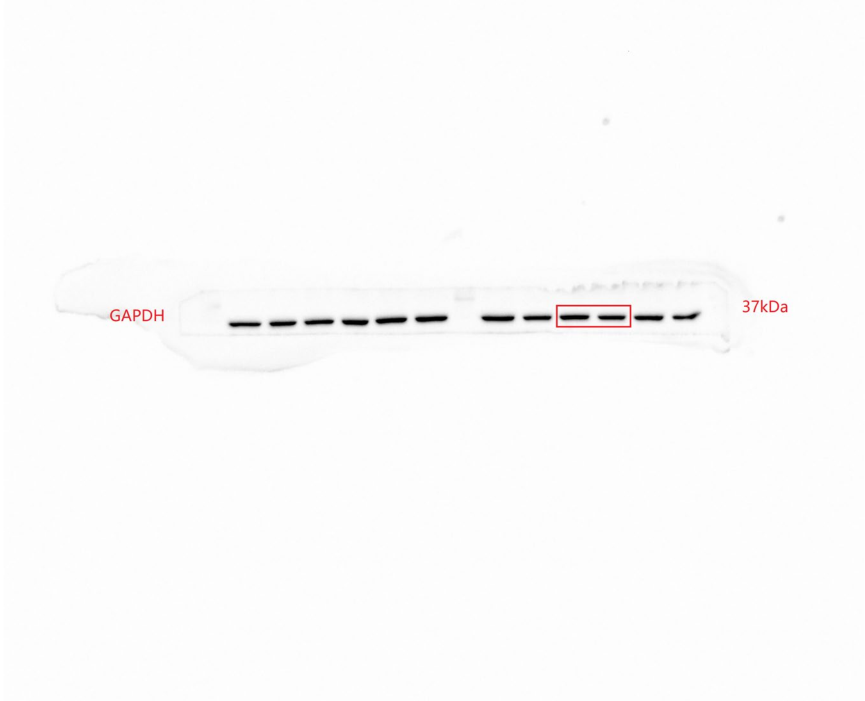

Supplementary files Fig 3. The LAMC2 and GAPDH protein bands are corresponding the original band of Fig. 2D in the manuscript.

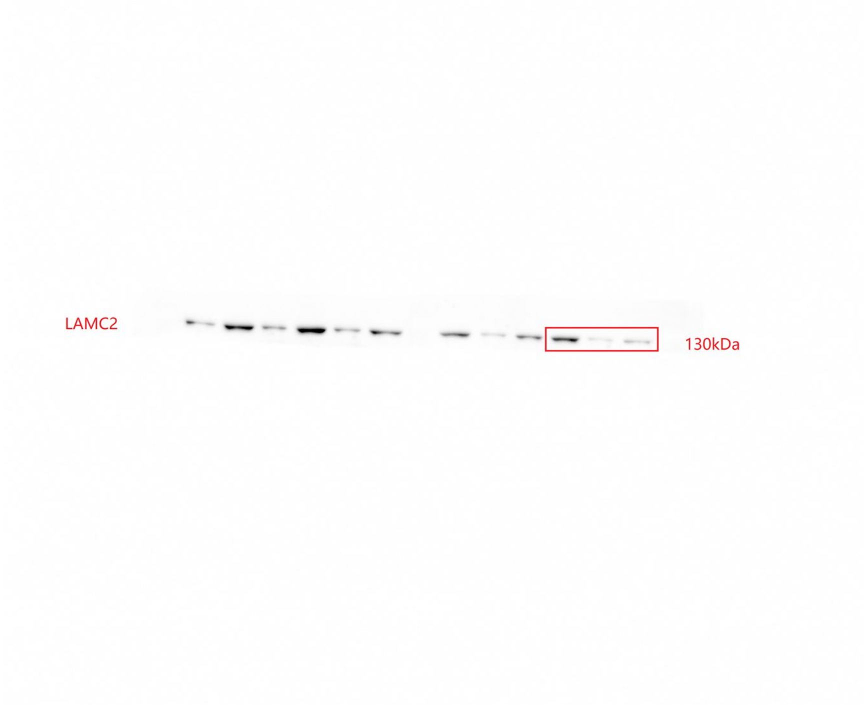

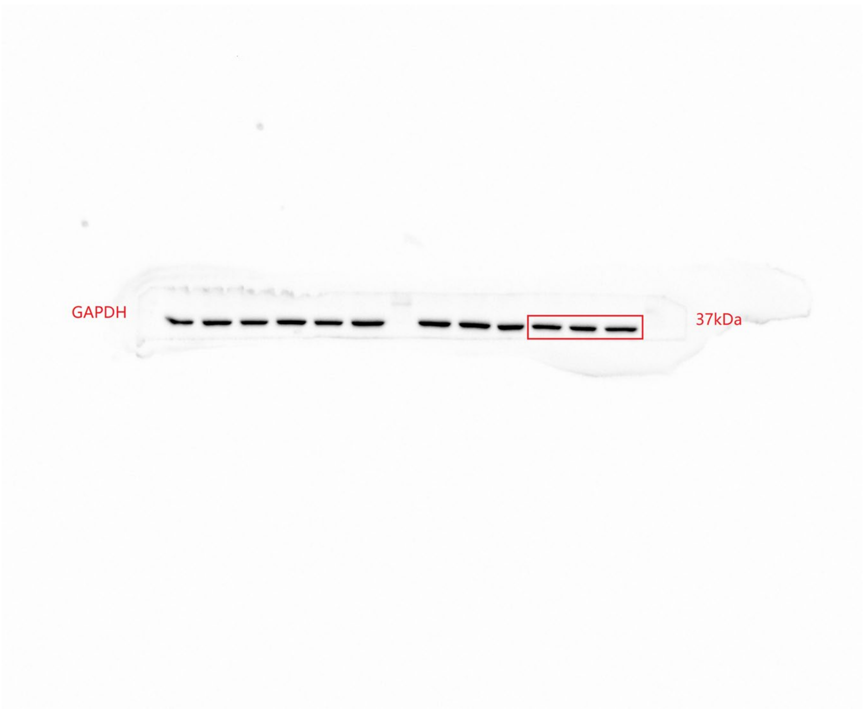

Supplementary files Fig 4. The LAMC2 and GAPDH protein bands are corresponding the original band of Fig. 2F in the manuscript.

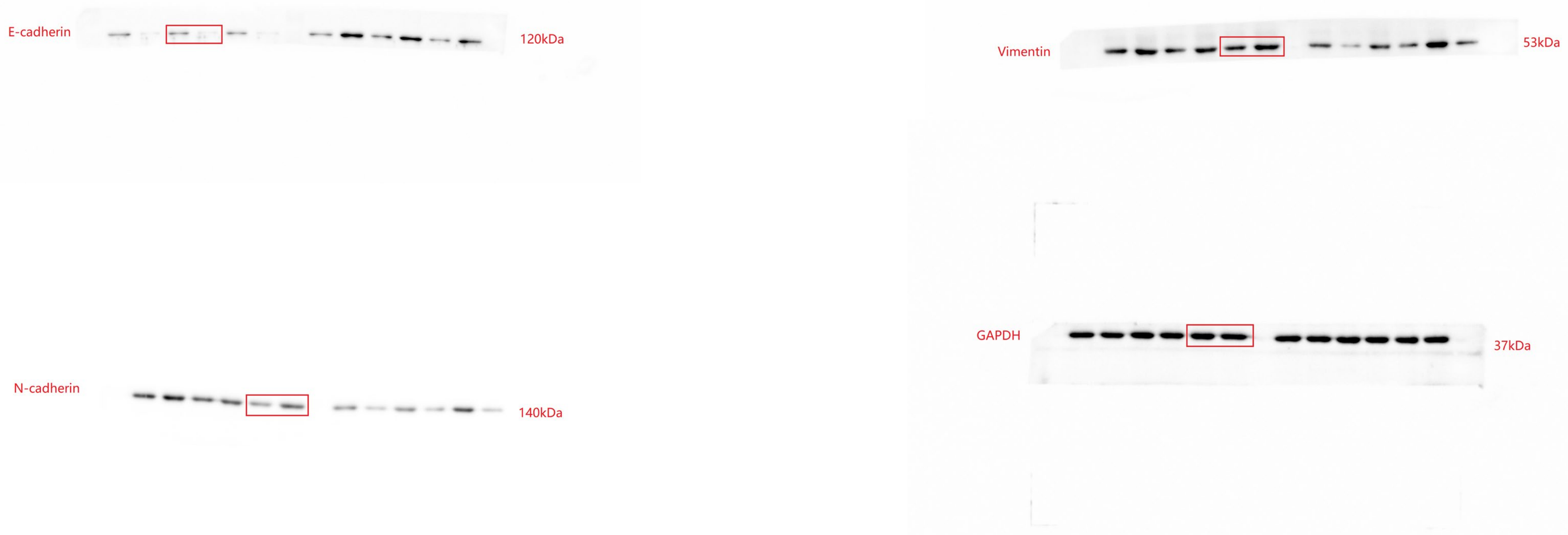

Supplementary files Fig 5. The The E-cadherin, N-cadherin, Vimentin and GAPDH protein bands are corresponding the original band of Fig. 5A in the manuscript.

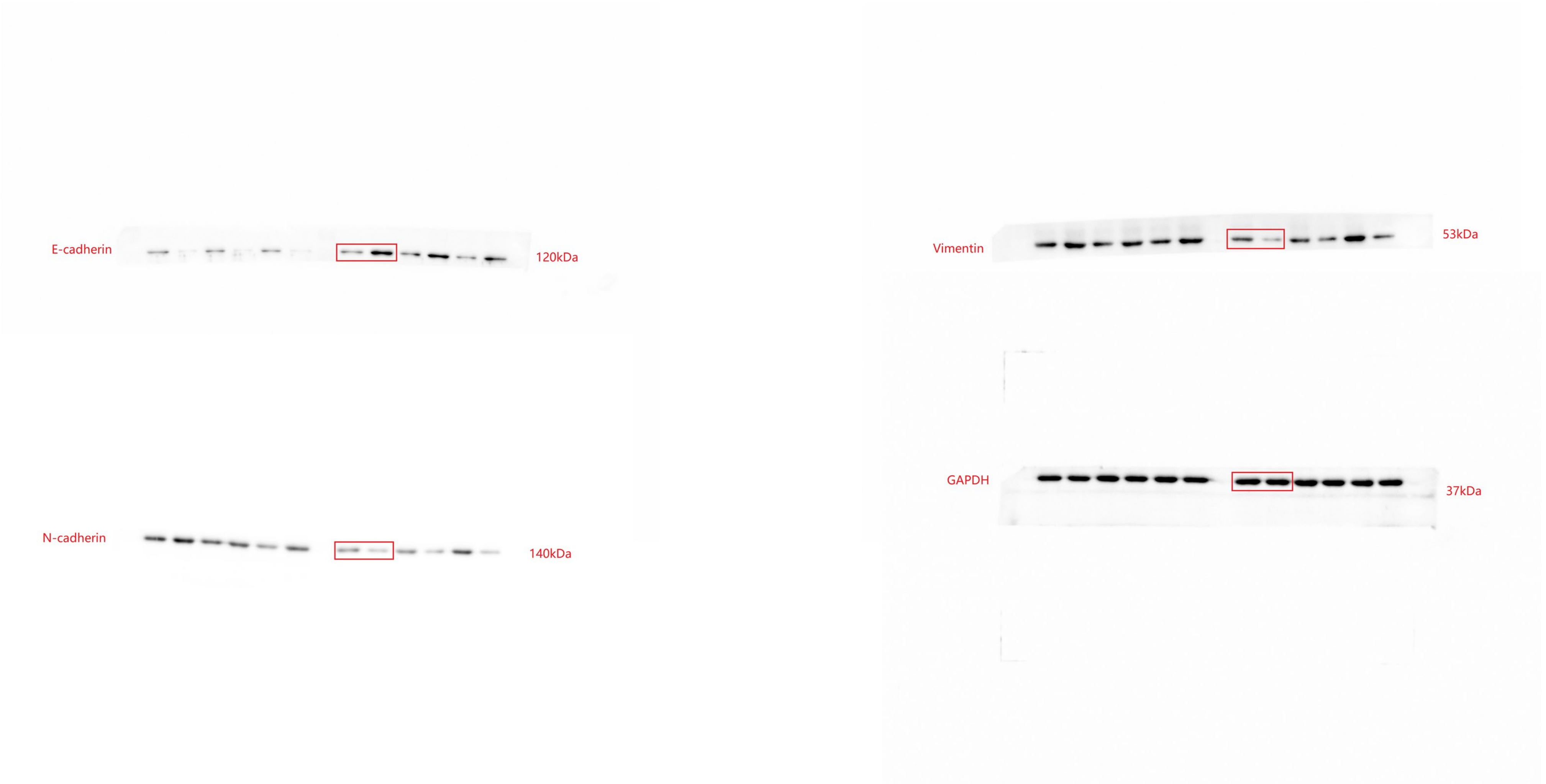

Supplementary files Fig 6. The E-cadherin, N-cadherin, Vimentin and GAPDH protein bands are corresponding the original band of Fig. 5B in the manuscript.

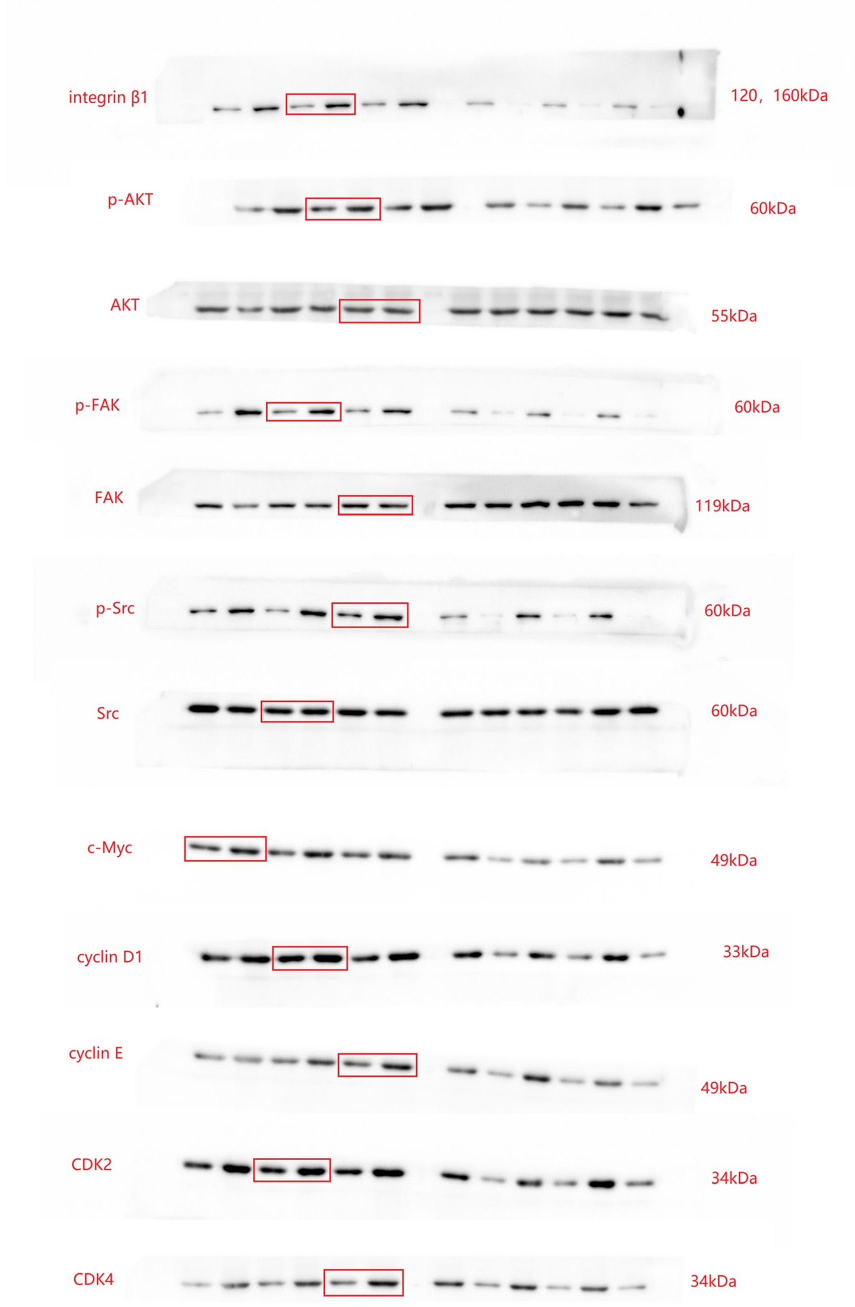

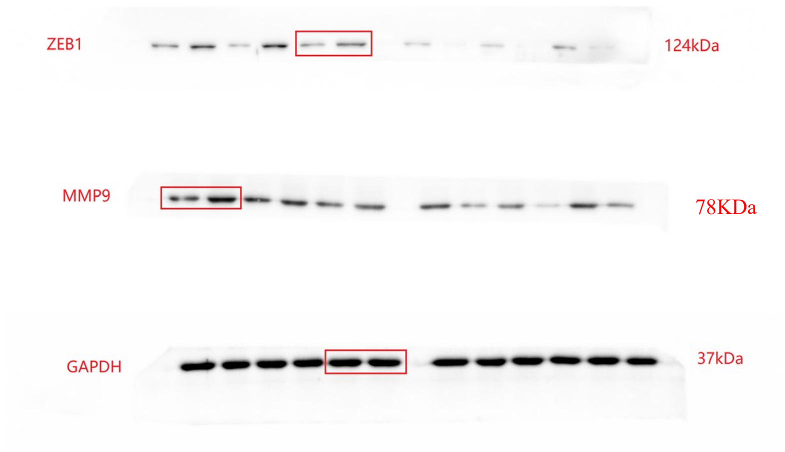

Supplementary files Fig 7. The integrinβ1, p-AKT, AKT, p-FAK, FAK, p-Src, src, c-Myc, cyclin D1, cyclin E, CDK2, CDK4, ZEB1, MMP9 and GAPDH protein bands are corresponding the original band of Fig. 6A in the manuscript.

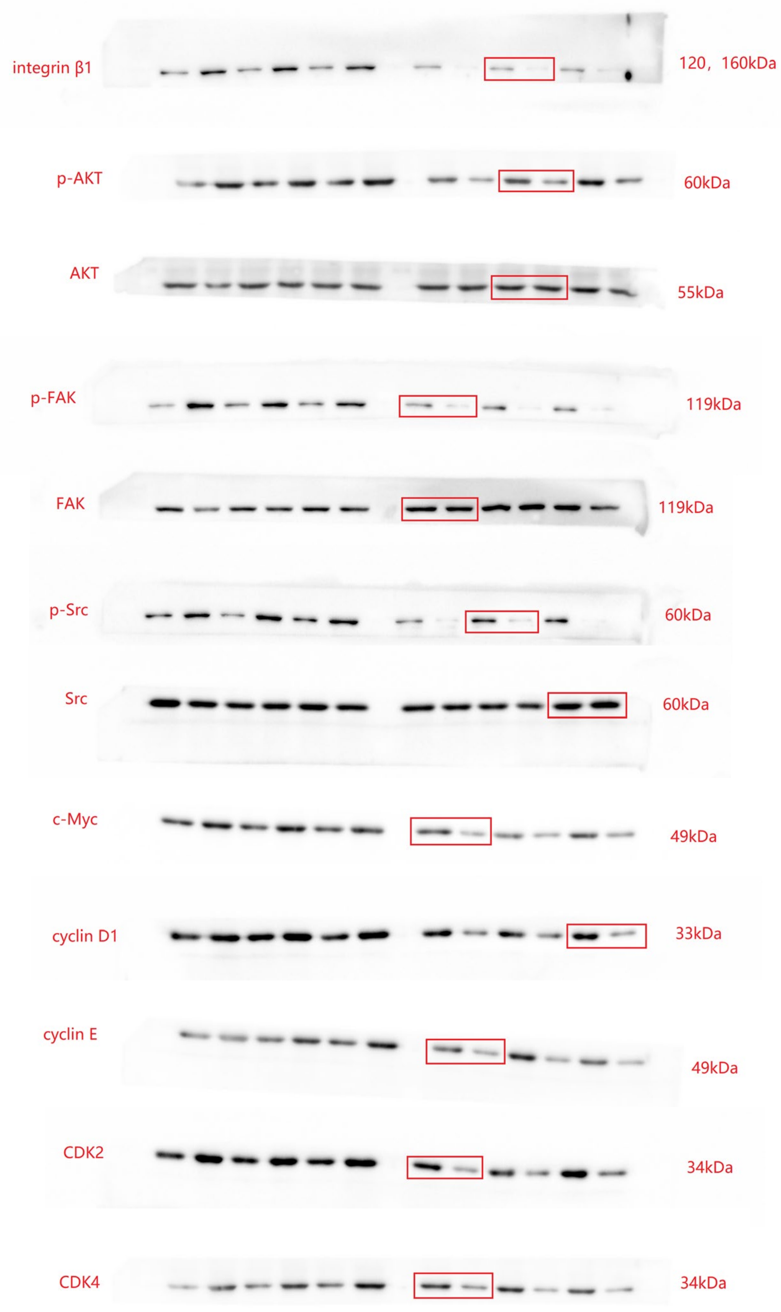

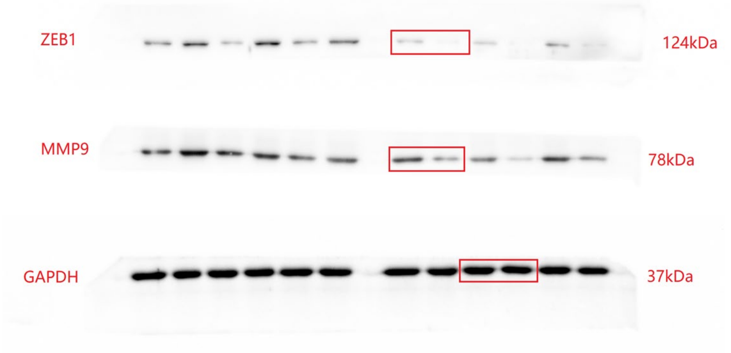

Supplementary files Fig 8. The integrinβ1, p-AKT, AKT, p-FAK, FAK, p-Src, src, c-Myc, cyclin D1, cyclin E, CDK2, CDK4, ZEB1, MMP9 and GAPDH protein bands are corresponding the original band of Fig. 6B in the manuscript.

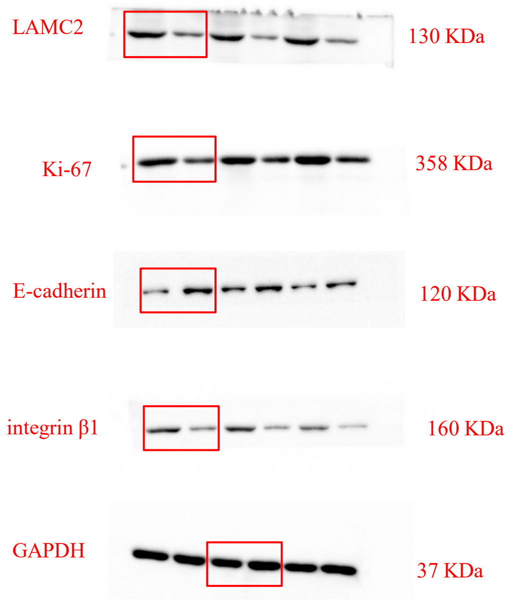

Supplementary files Fig 9. The LAMC2, Ki-67, E-cadherin and integrinβ1 protein bands are corresponding the original band of Fig. 8 in the manuscript.
